# Possible role of the cavernous sinus veins in cerebrospinal fluid absorption

**DOI:** 10.1186/1743-8454-4-3

**Published:** 2007-04-16

**Authors:** Miles Johnston, Dianna Armstrong, Lena Koh

**Affiliations:** 1 Neuroscience Program, Department of Laboratory Medicine and Pathobiology, Sunnybrook Health Sciences Centre, University of Toronto, 2075 Bayview Avenue, Toronto, Ontario, M4N 3M5, Canada

## Abstract

The purpose of this investigation was to enhance our understanding of cerebrospinal fluid (CSF) absorption pathways. To achieve this, Microfil (a coloured silastic material) was infused into the subarachnoid space (cisterna magna) of sheep *post mortem*, and the relevant tissues examined macroscopically and microscopically. The Microfil was taken up by an extensive network of extracranial lymphatic vessels in the olfactory turbinates. In addition however, Microfil also passed consistently through the dura at the base of the brain. Microfil was noted in the spaces surrounding the venous network that comprises the cavernous sinus, in the adventitia of the internal carotid arteries and adjacent to the pituitary gland. Additionally, Microfil was observed within the endoneurial spaces of the trigeminal nerve and in lymphatic vessels emerging from the epineurium of the nerve. These results suggest several unconventional pathways by which CSF may be removed from the subarachnoid space. The movement of CSF to locations external to the cranium via these routes may lead to its absorption into veins and lymphatics outside of the skull. The physiological importance of these pathways requires further investigation.

## Background

Microfil is a coloured, liquid silicone rubber compound. This agent facilitates the generation of three-dimensional images of the spaces into which it has been injected. In previous reports, we infused Microfil into the subarachnoid compartment of a variety of species including mice, rats, rabbits, pigs, sheep, monkeys and humans. We noted that this contrast agent was carried through the cribriform plate into an extensive network of lymphatic vessels in the nasal submucosa of these species [1]. These data supported *in vivo *physiological studies that demonstrated an important role for extracranial lymphatic vessels in CSF absorption [2-6]. Additionally, we also noted the presence of Microfil at locations not associated commonly with CSF transport pathways. In this paper, we present these findings and speculate on whether these unconventional anatomical locations have a role in cranial CSF absorption.

## Methods

Details of the methods can be found in our earlier publications [1,7]. Briefly, randomly bred sheep (n = 29; 2.6–35 kg) including newborns (2–7 days old) and adult animals (6–8 months old) were used for this investigation. The newborns were anaesthetized initially by mask administration of incremental concentrations of halothane from 0.5 to 3%. The adult sheep were anesthetized initially by intravenous infusion of 2.5% sodium Pentothal solution. Subsequently, 1–3% halothane was delivered through an endotracheal tube via an A.D.S.1000 or Narkomed 2 respirator for surgical maintenance. A laminectomy was performed on the upper regions of the spinal cord (generally between C1–C2) and the cisterna magna was cannulated with an angiocatheter or a silastic tube of appropriate size. A ligature was placed around the spinal cord and tightened to compress the spinal cord and meninges. This ligature prevented Microfil from passing into the spinal subarachnoid compartment during injection. Following sacrifice of the animals with an intravenous overdose of pentobarbital (euthanyl, Bimeda-MTC, Cambridge, ON), all experiments were performed by infusing yellow Microfil^® ^(MV-122, Flowtech, Mass) manually into the cisterna magna over 10–15 min using a syringe. In several sheep, the carotid arteries were catheterized before euthanasia and 20 ml of blue Microfil^® ^(MV-120, Flowtech, Mass) was infused into both arteries immediately after euthanasia. After storage overnight at 4°C, the heads were skinned, sliced in a sagittal orientation and fixed in 10% formalin. Sectioning of the fixed tissues was made in several orientations including coronal and sagittal. The tissues were viewed under a dissecting microscope (Leica M651, Wild Leitz; EMZ-TR, Meiji Techno; Fisher Stereomaster) and images were captured on a Nikon digital camera (Coolpix 995). For histological analysis, the fixed tissues were sliced and embedded in paraffin. Samples were sectioned at 4 μm thickness and stained with haematoxylin and eosin. Histological assessments were performed using a Motic Digital Microscope (DMB5) and images acquired using Motic Images Advanced 3.0 software (GENEQ Inc. Montreal, Canada). All animal experiments were approved by the ethics committee at Sunnybrook Health Sciences Centre and conformed to the guidelines set by the Canadian Council on Animal Care and the Animals for Research Act of Ontario.

## General findings

**Figure 1:**
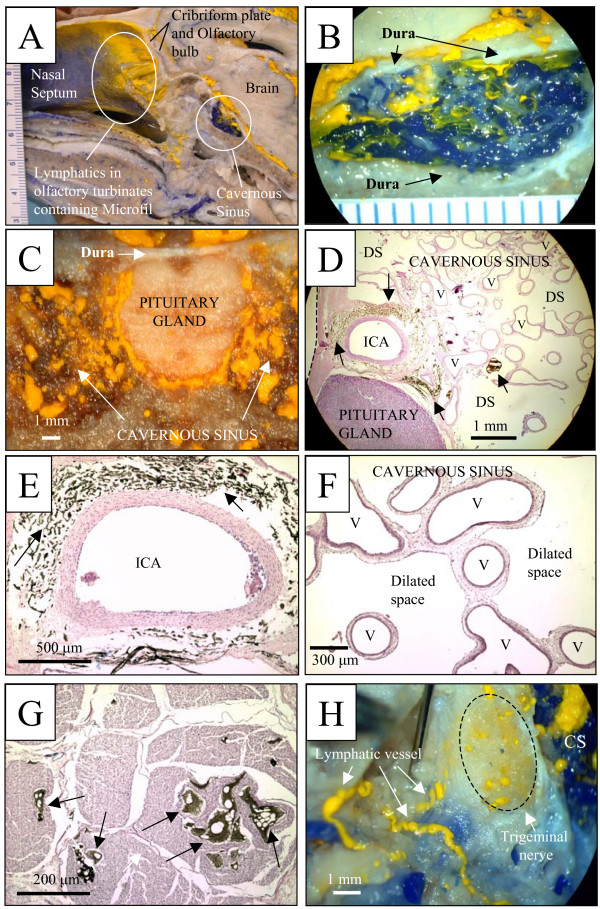
Microfil distribution patterns in the head of sheep. Reference scales are provided either as a ruler in the image (mm) or as a labelled longitudinal bar. A – Sagittal view of sheep head showing yellow Microfil in the lymphatic vessels associated with the olfactory turbinates and distributed within the cavernous sinus. B – Higher magnification of a sagittal view of the cavernous sinus demonstrating yellow Microfil from the subarachnoid space and blue Microfil in the venous plexus. The green colour indicates some mixing of the two coloured Microfils. C – Coronal section illustrating yellow Microfil distributed around the pituitary gland and within the cavernous sinus. D – Histological section of the cavernous sinus (the dura is delineated by a dashed line) showing yellow Microfil (dark brown) located in the connective tissue adventitia (black arrow) of the internal carotid artery (ICA). In addition, residual Microfil can be observed in the tissues surrounding the pituitary gland and within the spaces around the venous plexus of the cavernous sinus (black arrows). Most of the Microfil at the latter location has been lost during tissue processing. However, the dilated spaces (DS) presumed to originally contain the contrast agent, are clearly seen. Some of the venous channels are labeled (V). E – Enlargement of Fig 1D demonstrating Microfil deposition in the adventitia (black arrows) surrounding the internal carotid artery (ICA). F – Histological section showing the venous structure of the cavernous sinus. The spaces between the veins are dilated and are presumed to have contained Microfil that was lost on tissue preparation. Some of the venous channels are labeled (V). G – Histological section of a portion of the trigeminal nerve. Microfil (black arrows) can be observed within the endoneurium of the nerve. H – Cut surface of the trigeminal nerve (dashed circle) adjacent to the cavernous sinus (CS) showing yellow Microfil distribution within the nerve. Lymphatic vessels (white arrows) containing yellow Microfil can be seen emerging from the epineurium of the nerve.

The distribution of Microfil was similar in both newborn and adult sheep. Yellow Microfil was observed throughout the subarachnoid compartment associated with the base of the brain. In addition to the distribution of yellow Microfil in the lymphatic vessels in the olfactory turbinates that has been described in previous reports, a consistent finding in almost all animals that were perfused successfully, was that Microfil passed through the dura at the base of the brain and entered the cavernous sinus (Figs. 1A–C). The cavernous sinus is a venous plexus situated between the periosteal and meningeal dural layers. The sinus is composed of veins, arteries, meninges, and is traversed by several nerves including various cranial nerves: III (oculomotor), IV (trochlear), VI (abducens), and the ophthalmic branch of V (trigeminal) [8]. At a macroscopic level, yellow Microfil filled the spaces around the veins of the cavernous sinus (Figs. 1B,C) and enveloped the pituitary gland (Fig. 1C). When blue Microfil had been infused into the blood, it mixed with the yellow Microfil in the cavernous sinus creating a green colour (Fig. 1B). The yellow Microfil appeared to pass into the veins and in some preparations, the green mixture could be followed down to the level of the jugular vein.

During processing for histology, the yellow Microfil turned dark brown and most of it was removed from the tissues during tissue preparation. Nonetheless, some residual Microfil was observed where it was apparently trapped by connective tissues. The yellow contrast agent was noted in the adventitia of the internal carotid arteries and was associated with the tissues around the pituitary gland (Figs. 1D,E). The spaces around the veins of the cavernous sinus that were presumed to contained yellow Microfil originally, were greatly distended (Fig. 1F). Additionally, yellow Microfil was observed within the endoneurium of the trigeminal nerves (Fig. 1G) and when observed macroscopically, was present in lymphatic vessels emerging from the epineurium of the nerve (Fig. 1H).

We recognize the need to be cautious in extrapolating physiological concepts from anatomical studies employing Microfil, particularly because the Microfil is infused *post mortem*. Nonetheless, Microfil distribution patterns have proven to be useful in the past to elucidate lymphatic CSF absorption pathways and might provide insights into several other 'unconventional' pathways by which CSF might exit the subarachnoid compartment to reach extracranial sites. These potential routes include CSF entry into dura, CSF passage along the adventitia of blood vessels, CSF transport within nerves and CSF movement through dura into the peri-venous spaces associated with the cavernous sinus.

### CSF Transport into Dura

Regarding the movement of CSF into dura, we observed that yellow Microfil passed through the dura at the base of the brain although in this case, it seemed to do so only in association with the cuff of adventitia of the internal carotid artery. In contrast, in a previous report we noted that yellow Microfil coloured the dura proper in the vicinity of the superior sagittal sinus [9]. If the movement of CSF into dural or adventitial connective tissues occurs under physiological conditions, it is likely that blood capillaries or lymphatic vessels within the dura absorb this fluid. In this regard, lymphatics have been identified in the dura at the base of the skull in dogs and these vessels have connections with the downstream cervical lymphatics [10]. In rats, lymphatics exist around the wall of the sagittal sinus, in the areas of the confluence of sinuses in proximity to the mesothelial cells of the subdural spaces, and close to the vasculature of the dural tissues [11].

There is however, at least one theoretical objection to a possible role for dural lymphatics in CSF drainage. The cellular architecture and the presence of tight junctions between arachnoid cells are believed to contribute to the blood-brain/CSF barrier [12]. Without this barrier function, the extravasated fluid and solutes from the permeable dural capillaries would enter the dura interstitium and possibly gain access to CSF. However, for any dural CSF transport to occur, presumably CSF would have to pass through the supposed barrier provided by the arachnoid membrane to enter dural tissues. Such transport is thought to occur through specialized areas of the membrane where it projects into the venous sinuses (arachnoid villi and granulations). CSF transport through the arachnoid membrane at other locations has not been given much consideration although there is some evidence to support this concept. In the studies by Killer et al., India ink injected into the subarachnoid space of the optic nerve penetrated the arachnoid and entered the interstitial compartment and lymphatics in the dura of the nerve [13]. There is the suggestion that transport across the arachnoid membrane in this tissue may be in part vesicular [14]. Additionally, radiolabeled albumin injected into the subdural space in rabbits was observed to enter plasma [15] and it seems likely that dural lymphatics contributed to this clearance.

### Blood Vessel Adventitial – CSF Transport

There is support for the view that parenchymal interstitial fluid or CSF can be carried through the adventitia of cerebral blood vessels [16-18]. It would appear that the collagen-rich perivascular spaces can be traced through the skull base in association with the internal carotid artery and these conduits are believed to form potential channels for the drainage of CSF to the cervical lymph nodes in the neck region. However, one group contends that the channels do not extend to the extracranial carotids and jugulars [19]. Whatever the final destination of the adventitial CSF, the Microfil images imply that some of the CSF that passes through the dura at the base of the brain around this location may end up in the peri-venous spaces of the cavernous sinus.

### CSF Transport along Nerves

The fact that CSF can move along the outer surface of nerves as they leave the brain and spinal cord is well established in the literature [1,7,19,20]. In addition, in past studies we observed that several cranial and spinal nerves contained Microfil located within longitudinal channels in the endoneurial tissues [7]. In the investigation reported here, we noted a similar phenomenon within the portion of the trigeminal nerve associated with the cavernous sinus. We also noted that lymphatic vessels emerging from this nerve contained Microfil. Since several other nerves traverse the cavernous sinus, it is also possible that these nerves could provide a thru-way from the basal subarachnoid space and cisterns to the extracranial tissue, but this was not investigated.

In the literature, there is some support for the concept that the CSF compartment and extracranial lymphatic vessels are connected by fluid-filled spaces within the nerves. For example, horseradish peroxidase injected intrathecally was observed in epineural, perineural and endoneural spaces and extended beyond the epineurium into lymphatics [21]. In addition, physiological data would seem to support the proximo-distal convection of endoneurial fluid [21,22]. Measurements of endoneurial fluid pressure indicate proximo-distal gradients [23] and there is a close relationship between CSF pressure and interstitial fluid pressure within certain facial nerves [24].

### CSF transport into Cavernous Sinus

One of the most consistent findings was the appearance of Microfil in the peri-venous spaces of the cavernous sinus. The existence of the contrast agent at this location may have been due to movement through the dura, passage along the adventitia of blood vessels, or possibly associated with transport along the various nerves which enter and leave this space. To the best of our knowledge, there have been no previous reports of CSF movement into the cavernous sinus.

The complex formed by the superior sagittal sinus and arachnoid villi and granulations is the most well studied interface between CSF and venous blood. Absorption of CSF from the subarachnoid space across the arachnoid projections into the sinus is assumed to be driven by the hydrostatic pressure difference between the two compartments. Blood vessels and nerves do not transect the dura of the superior sagittal sinus (as they do the cavernous sinus). Accordingly, perivascular and endoneurial spaces are not part of the hypothesized CSF absorption system at this location.

While there has been a report of the presence of arachnoid projections in the cavernous sinus, their physiological significance has never been elucidated [8]. However, a consideration of the Starling forces across the thin-walled veins in the cavernous sinus, suggest a theoretical mechanism by which, significant amounts of free water (originating as CSF) may be absorbed directly into the venous plexus. The Starling equation assumes two driving forces, the hydrostatic and osmotic pressure gradients, and a filtration coefficient, which sets the conductance to flow. In venules and veins, the hydrostatic pressure is much lower than mean capillary pressure (which is about 25 mm Hg) and is probably much lower than the opposing osmotic pressure gradient (which in most normal systems is around 20–25 mm Hg). This situation would favor CSF (or interstitial fluid) absorption into the venules and veins. The filtration coefficient of cerebral microvessels (i.e. of the blood-brain barrier) is very small but is likely much larger for the vessels within the cavernous sinus. Rapid fluid transfer between the two compartments would readily occur if the filtration coefficient for the walls of these veins approaches those of the microvessels within other non-central nervous system tissues.

## Conclusion

In summary, the presence of intracisternally-administered Microfil external to the dura at the base of the brain suggests several ways in which CSF may gain access to potential absorption sites. The interstitium of the dura, adventitia of blood vessels and endoneurium of nerves might provide a link between the subarachnoid space and extracranial tissues. However, unless evidence is obtained to the contrary, it seems likely that these 'conduits' are of only minor significance to the volumetric clearance of CSF from the brain. From a volumetric perspective, absorption of CSF into the veins of the cavernous sinus would appear to have greater potential, a concept that appears to warrant further investigation.

## Competing interests

The author(s) declare that they have no competing interests.

## Authors' contributions

MJ: conceived of the study, and participated in its design and coordination.

DA: assisted in all experiments and helped in the preparation of the manuscript.

LK: helped in the preparation of the manuscript.

All authors read and approved the final manuscript.
